# Observations on a Mode of Annihilating the Pains Which Follow Surgical Operations

**Published:** 1849-06

**Authors:** Jules Roux

**Affiliations:** Commissioners, Flourens, Roux, Velpeau


### BUFFALO MEDICAL JOURNAL

AND

### MONTHLY REVIEW.

**VOL. 5.** JUNE, 1849. **NO. 1.**

### ORIGINAL COMMUNICATIONS.

ART. 1.—*Observations on a mode of Annihilating the pains which follow Surgical Operations.* By JULES ROUX. (Commissioners, Flourens, Roux, Velpeau.) Translated by JAMES BRYAN, M. D. Professor of Surgery in Geneva Medical College.

The pains produced by Surgical operations may be divided into three classes: 1st. The pains of the operation: 2d. Those which immediately follow the operation: 3rd. Consecutive pains, or those which take place during the stage of cicatrization. Distinct in their causes, their intensity, their duration, and the time they continue, these pains do not differ in the mode by which they are relieved. This mode, which completes the American discovery, I have found, if I mistake not, in *direct etherization,* to the wounded surfaces.

Local and direct etherization, which does not appear to differ from indirect and general, except that the blood is not the medium of communication or which employed, consists in placing an anesthetic fluid for five, ten or fifteen minutes in contact with the wound. This application is made by means of a pencil, a pledget of charpie, a spunge, a sack containing ether, if the fluid is employed in the state of vapor, or, what is preferable, by moistening the wound, or filling it with the anesthetic agent. To the present time my attention has been directed only to the aldehyde, the ether, and the choloform, the latter particularly was used in nearly all the cases referred to in these remarks.

In the first series of experiments, I subjected to direct etherization small recent wounds, as well as the more considerable ones, the operations for phymosis, amputations of the fingers, the leg, and the fore-arm, and have made the following observations:

Where the patients have not been previously subjected to the influence of ether through the lungs, they experience a sensation of pricking, smarting, of heat, of sharp pain, and even of burning. After a few moments these phenomena diminish, rapidly disappear, and give place to partial etherization, limited to the parts directly etherized: local insensibility for forty-eight hours at least, keeps the patients in the most complete comfort.

When, on the other hand, the patients have undergone etherization, and, before returning to general sensibility, the wounds are exposed to the local influence of ether, the pains from the immediate action of the anesthetic agent are not experienced, and the patient, entirely composed, passes through the first two or three days after an operation, without pain.

Local anesthesia produced by direct etherization continues, thus, long enough to annihilate the pains of the second class. I proceed, in support of this assertion, to state the last case of amputation which I have subjected to direct etherization.

On the 8th of November, 1848, I performed on F. Vaslot, aged thirty years, an amputation of the fore-arm. After the separation of the limb, and the application of four ligatures, the stump was washed with choloform, while general etherization with choloform was produced. The stump, without dressing even, was placed (as is done by Sedellot) on a cushion, and covered with a piece of linen.

During two days Vaslot declared he did not experience the least pain in the wound, after which, at intervals, he began to experience it very slightly. Although the patient had been affected both in the right eye and the fore-arm of the same side, he had no fever, slept well, the appetite good, and the progress of the wound regular. At this time, eleven days since the operation, cicatrization, already accomplished on the margins of the wound, is almost finished.

The fact of the local insensibility of traumatic surfaces, without shock, or general insensibility, with the fact that the system is freed from pain during the first days after the great operations, and that this condition continues up to the moment when cicatrization commences, appear to me to be of sufficient importance to engage the attention of Surgeons in the matter.

In a second series of experiments, I tried to annihilate pains of the third class, or those which may occur during the restoration of the tissues. I have often accomplished this by direct etherization.

I think I have observed, with many other Surgeons, that, after general etherization, the second order of pains have less intensity, and a shorter duration than when we have neglected to apply the powerful means of anesthesia. I have remarked, also, that after direct etherization, the pains of the third class are considerably ameliorated. They were nothing in the patient who had suffered a partial amputation of the finger: and nearly nothing, or so light in the cases of amputation of the leg and fore-arm, that I did not think it worth while to apply consecutive anesthesia.

On the other hand the pains of separating wounds may be calmed, and even annihilated by the local application of choloform, and the application, far from injuring these surfaces, appears, on the contrary, to exert a happy influence upon them. I have frequently shown to the Surgeons of the Marine, attached to the service of which I have charge, inguinal ulcerations, following syphilic eruptions, insensible to the nitrate of silver, when its application before direct etherization, induced severe pains for several hours. These facts go to show, that etherization will overcome the pains of the third class, in traumatic surfaces, while its action is less certain than in the preceding cases.

In the course of my labors, I have not observed, without astonishment, that chloroform, which so painfully reddens the skin, with or without the cuticle, without completely deadening sensibility, produces, on the contrary, on traumatic surfaces, with an evanescent irritation, complete insensibility. The reason for this difference is entirely anatomical.

Direct etherization, for which I prefer the liquid chloroform to that in vapor, is remarkable for its harmlessness; I have never regretted having recourse to it. In no case, even when I have injected sixteen grammes of chloroform into the tunica vaginalis of a patient affected with hydrocele, have I observed appreciable phenomena, which could be attributed to the absorption of the fluid. I have always been struck with the calmness of the patients, the absence of febrile action, and with the simplicity and regularity of the progress of suppurating wounds.

If the benefits of local anesthesia, which a few facts alone have confirmed, should receive the sanction of experience, we may hope that direct etherization will comprehend in its application, all the conditions of traumatic wounds.

The statistics of amputations performed with the aid of etherization, has, by a great number of cures, shown all the advantages which may be obtained by anesthesia, in the great operations of surgery. May we not, hence, infer, that direct etherization to traumatic surfaces will add something more to these happy results?

